# PHYSICAL ACTIVITY AND THE DEVELOPMENT OF DEMENTIA BETWEEN AGES 70 AND 95

**DOI:** 10.1093/geroni/igad104.0412

**Published:** 2023-12-21

**Authors:** Irit Stessman-Lande, Jochanan Stessman, Jeremy Jacobs

**Affiliations:** Faculty of Medicine, Hebrew University of Jerusalem, and Hadassah Medical Center, Israel, Jerusalem, Yerushalayim, Israel; Hebrew University of Jerusalem, and Hadassah Medical Center, Israel, Jerusalem, Yerushalayim, Israel; Faculty of Medicine, Hebrew University of Jerusalem, and Hadassah Medical Center, Israel, Jerusalem, Yerushalayim, Israel

## Abstract

Whilst the protective effects of physical activity (PA) on cognitive function are well documented, it remains unclear if the preventive effect continues irrespective of advancing age. This study examines the association between physical activity and subsequent dementia between ages 70-95. The Jerusalem Longitudinal Study (1990-2023) prospectively follows a representative community-dwelling cohort born 1920-21, assessed at home visit during 1990,1998, 2005, 2010, 2015 at ages 70, 78, 85, 90, and 95 (n=430, 715, 1136, 660, 492) respectively. Comprehensive assessment included Mini Mental State Examination (MMSE) defining dementia (≤23/30), and PA assessment, dichotomized to Active (PA-A) (>4hours a week) versus Sedentary (PA-S) (< 4 hours weekly). Logistic regression analyses determined Odds Ratios (OR) for developing dementia, adjusted for baseline MMSE, gender, education, depression and BADL. At ages 70, 78, 85, 85, 90, and 95 the frequency of PA-A was 45.9%, 69.7%, 48.7%, 9.5%, 11.9%; and frequency of dementia among PA-A vs. PA-S was 1.7% vs. 5.0%, 1.7% vs. 12.6%, 10.6% vs. 48.5%, 8.6% vs. 35.5%, 18.3% vs. 29.9% (all p< 0.0001 except age 70 due to small numbers) respectively. Adjusted ORs ratios among PA-A at baseline for subsequently developing dementia between ages 78-85, 85-90, 90-95 were: OR 0.44 (95%CI 0.20-0.98); OR 0.52 (95%CI 0.30-0.90); OR 0.37 (95%CI 0.13-1.07) respectively. Our findings show that physical activity is associated with reduced development of dementia throughout follow-up between ages 70-95, and support the hypothesis that there is no upper age-limit to the beneficial effects of PA on cognition.

